# Coordinate PI3K pathway and Bcl-2 family disruption in AML

**DOI:** 10.18632/oncotarget.809

**Published:** 2012-12-31

**Authors:** Prithviraj Bose, Mohamed Rahmani, Steven Grant

**Affiliations:** Massey Cancer Center, and the Departments of Internal Medicine (Division of Hematology/Oncology), Virginia Commonwealth University, Richmond, VA; Massey Cancer Center, and the Departments of Internal Medicine (Division of Hematology/Oncology), Virginia Commonwealth University, Richmond, VA; Massey Cancer Center and the Departments of Internal Medicine (Division of Hematology/Oncology), Microbiology and Immunology, Biochemistry and Molecular Biology, Human and Molecular Genetics, and the Institute for Molecular Medicine, Virginia Commonwealth University, Richmond, VA

The B-cell lymphoma-2 (Bcl-2) family of pro- and anti-apoptotic proteins controls the mitochondrial pathway of apoptosis. Their central role in mediating the final common pathway of apoptosis in response to internal and external stressors makes them attractive therapeutic targets in neoplastic cells, which are often “primed” for apoptosis. The major anti-apoptotic proteins are Bcl-2, Bcl-xL and myeloid cell leukemia-1 (Mcl-1). These promote cellular survival by sequestering pro-apoptotic proteins which function as apoptosis “sensitizers” (e.g., Bim, Bad) or “effectors” (Bax, Bak). Multiple studies have implicated Bcl-2 family proteins in AML pathogenesis, prognosis and resistance to chemotherapy.

The discovery of the “BH3-mimetic” ABT-737, a specific inhibitor of the anti-apoptotic actions of Bcl-2 and Bcl-xL, demonstrated for the first time that specific protein-protein interactions could be disrupted using a small molecule [1]. Several groups [2, 3] have shown that Mcl-1, which is not inhibited by ABT-737, is the primary determinant of resistance to this agent. This, along with recent evidence that Mcl-1 may be more critical to the development and maintenance of AML than Bcl-2 or Bcl-xL [4], has prompted interest in combining agents that down-regulate Mcl-1 with ABT-737 in AML to simultaneously disable multiple arms of the mitochondrial apoptotic regulatory machinery. Thus, cyclin-dependent kinase inhibitors [3] and sorafenib [5] synergize with ABT-737 in pre-clinical studies of AML.

The phosphatidylinositol-3-kinase (PI3K)/Akt/mammalian target of rapamycin (mTOR) pathway is one of the most frequently dysregulated survival pathways in human malignancies, including AML, leading to its emergence as a therapeutic target in this disease [6]. For example, internal tandem duplication mutations in the *FLT3* gene, affecting approximately 25% of patients with AML, lead to constitutive FLT3 signaling which activates Akt and confers a poor prognosis. Similarly, *Ras* and *KIT* mutations, common in core-binding factor AML, activate the PI3K/Akt/mTOR pathway. In many cases, the basis for addiction to this pathway, manifested by basal Akt activation, remains unknown. While mTOR inhibitors are approved for advanced renal cell carcinoma and the PI3K delta inhibitor GS-1101 (CAL-101) appears promising in CLL, a new class of agents that inhibit *both* PI3K and mTOR signaling (e.g., NVP-BEZ235, GDC0980) may offer additional therapeutic advantages in AML [7].

In light of the above considerations, and the known ability of PI3K inhibitors to down-regulate Mcl-1 and up-regulate/activate the pro-apoptotic proteins Bim, Bad, Bax and Bak, the effects of dual PI3K/mTOR and Bcl-2/Bcl-xL inhibition in AML cell lines, patient-derived leukemic blasts, and xenograft models of AML were recently examined [8]. Notably, tet-inducible short hairpin RNA constructs directed against Akt or Bcl-2 and Bcl-xL individually or together, and studies employing pharmacologic inhibitors (e.g., NVP-BEZ235/PI-103 and ABT-737), revealed that the PI3K/Akt/mTOR pathway and Bcl-2/Bcl-xL play important coordinate roles in protecting leukemia cells from lethality. Specifically, genetic or pharmacologic interventions simultaneously inhibiting the PI3K/Akt/mTOR pathway and disabling Bcl-2/Bcl-xL dramatically enhanced leukemic cell death, with minimal toxicity toward normal CD34^+^ hematopoietic progenitors. These findings were recapitulated *in vivo* in an AML xenograft model with little toxicity, likely reflecting both a dependence of leukemic cells on the PI3K/Akt/mTOR pathway for survival, and relative sparing of normal cells by BH3-mimetic treatment. Mcl-1 down-regulation resulted from PI3K/mTOR inhibition, at least in part through GSK3 activation, which promotes the proteasomal degradation of Mcl-1. Although BEZ235 alone down-regulated Mcl-1, it did not significantly trigger cell death, underscoring the importance of concomitant Bcl-2/Bcl-xL inhibition, as all three major anti-apoptotic proteins sequester the apoptosis effectors Bax and Bak. Significantly, release of Bax and Bak from Mcl-1, Bcl-2 and Bcl-xL was observed.

Another novel finding was a marked increase in Bim binding to Bcl-2 and Bcl-xL accompanying PI3K/mTOR inhibition, a phenomenon abolished by ABT-737. Presumably Bim, liberated from these two anti-apoptotic proteins, as well as Mcl-1 (due to down-regulation), triggered apoptosis by activating Bax and Bak. A summary of these interactions is presented in Figure 1. A particularly intriguing observation was an apparent correlation between responses to combined treatment with PI3K/mTOR inhibitors and ABT-737 and baseline Akt activation in primary AML blasts, raising the possibility that such cells may be addicted to this pathway and particularly vulnerable to its interruption. In fact, it is conceivable that assessment of Akt pathway activation may be a more reliable predictor of susceptibility to a dual PI3K/mTOR blockade and BH3-mimetic strategy than mutations in related proteins. Clearly, definitive answers to this question will require analysis of a considerably larger number of specimens. In any case, a major thrust of recent therapeutic approaches to AML has been to tailor drug therapy to specific genetic mutations, e.g., those in *FLT3* or *KIT*. However, the success of this approach is uncertain due to the upstream location of these mutated oncoproteins and the marked redundancy of signal transduction pathways. Pending more definitive validation, the present findings suggest novel candidate biomarkers (e.g., basal pathway activation) that might better help realize the goal of personalized therapy for AML.

**Figure 1:**
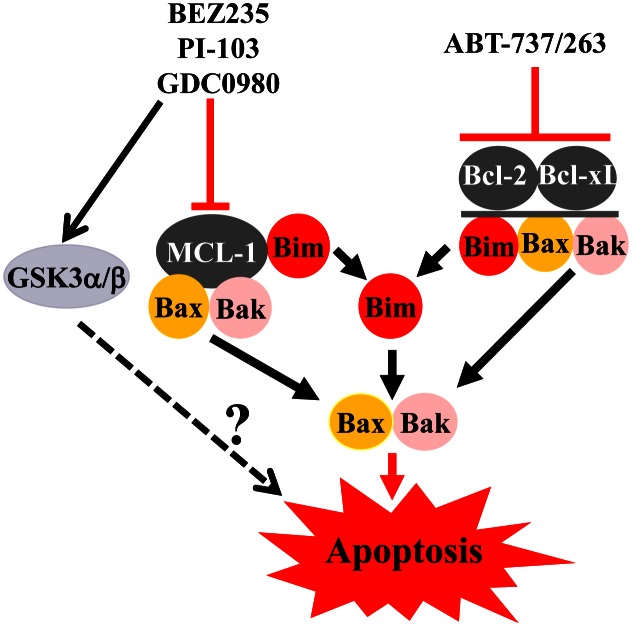
Model of interactions between PI3K/AKT/mTOR pathway inhibitors and Bcl-2 antagonists PI3K/mTOR inhibitors downregulate Mcl-1, at least in part through a GSK3-dependent mechanism, leading to the release of Bax, Bak and Bim, and a significant increase in liberated Bim binding to Bcl-2 and Bcl-xL. The latter phenomenon is completely abrogated by ABT-737, which concomitantly triggers the release of Bax and Bak from Bcl-2 and Bcl-xL. Thus, combined treatment leads to the release of all major proapoptotic Bcl-2 members (Bim, Bak and Bax), culminating in apoptosis induction. Finally, activation of GSK3 by Akt inhibition may also promote apoptosis through yet to be defined mechanisms.

## References

[1] Oltersdorf T (2005). Nature.

[2] Konopleva M (2006). Cancer Cell.

[3] Chen S (2007). Cancer Res.

[4] Glaser SP (2012). Genes Dev.

[5] Zhang W (2008). Leukemia.

[6] Martelli AM (2010). Oncotarget.

[7] Tamburini J (2008). Blood.

[8] Rahmani M (2012). Cancer Res.

